# Women and barriers to harm reduction services: a literature review and initial findings from a qualitative study in Barcelona, Spain

**DOI:** 10.1186/s12954-020-00429-5

**Published:** 2020-10-19

**Authors:** Sam Shirley-Beavan, Aura Roig, Naomi Burke-Shyne, Colleen Daniels, Robert Csak

**Affiliations:** 1 Harm Reduction International, 61 Mansell Street, London, E1 8AN UK; 2 Metzineres. Environments of Shelter for Womxn Who Use Drugs Surviving Violence, c/o ICEERS, Carrer de Sepúlveda, 65, Oficina 2, 08015 Barcelona, Spain

**Keywords:** Harm reduction, Women, Prison, Europe, Barcelona, Spain, Gender-based violence

## Abstract

**Background:**

There are an estimated 3.2 million women who inject drugs worldwide, constituting 20% of all people who inject drugs. The limited data that are available suggest that women who inject drugs are at greater risk of HIV and viral hepatitis acquisition than men who inject drugs. This increased vulnerability is a product of a range of environmental, social and individual factors affecting women, which also affect their ability to engage in health promoting services such as harm reduction.

**Methods:**

The researchers undertook a narrative literature review examining access to harm reduction services for women who use drugs in Europe and conducted semi-structured focus groups with women who use drugs and harm reduction and prison health workers in Barcelona, Spain.

**Results:**

Women who use drugs face multiple barriers to accessing harm reduction services. These include stigma, both in society in general and from health and harm reduction workers in prisons and in the community; gender-based violence and a lack of services that are equipped to address the interaction between drug use and experiences of violence; criminalisation in the form of legal barriers to access, arrest and harassment from law enforcement, and incarceration; and a lack of services focused on the specific needs of women, notably sexual and reproductive health services and childcare. In Barcelona, participants reported experiencing all these barriers, and that their engagement with the Metzineres harm reduction centre had to some extent mitigated them. However, women continued to experience structural barriers to harm reduction service access.

**Conclusions:**

Women and gender non-conforming people who use drugs face unique barriers to accessing harm reduction services. While services such as Metzineres can be life changing and life affirming for its members, it is incumbent on states to act to address the structural barriers to health faced by women who use drugs.

## Introduction

There are an estimated 3.2 million women who inject drugs worldwide, constituting 20% of all people who inject drugs [1]. Accounting for the concealing effects of criminalisation, gender power imbalances and stigma, this number is likely to be an underestimate [2]. The limited data that are available suggest that women who inject drugs are at greater risk of HIV and viral hepatitis acquisition than men who inject drugs [3]. This increased vulnerability is a product of a range of environmental, social and individual factors affecting women, which also affect their ability to engage in health promoting services such as harm reduction.

Health inequities occur where there are preventable differences between groups in access to services and resources that improve health outcomes [4]. Women are demonstrably subject to health inequities with regard to access to harm reduction services. Despite a clear need for harm reduction services for women, they continue to be dominated by “masculinist” concerns and do not meet women’s needs [5], and the literature indicates that women who use drugs are rarely adequately represented in design and evaluation of harm reduction services [6–8].

In Europe, the European Monitoring Centre for Drugs and Drug Addiction (EMCDDA) indicates women make up approximately a quarter of all people with drug dependence^1^ and around one-fifth of all entrants to drug treatment in Europe. EMCDDA reports women are particularly likely to experience stigma and economic disadvantage and to have less social support, among other factors [10].

A number of subgroups of women who use drugs have special needs. These subgroups, which often overlap, include pregnant and parenting women; women involved in sex work, who may often experience violence and stigma; women from ethnic minorities; women who may have been trafficked; and women in prison [11–13].

This paper builds on previous literature reviews that have examined the relationship between drug use and HIV among women [14–20], treatment effectiveness among women [21–24], stigma faced by women who use drugs [25–27], and drug use and intimate partner violence [28–31]. It seeks to build on this body of work by examining the literature on women and harm reduction services globally, with the specific aim of identifying the key barriers standing in the way of women’s access to these services. The paper categorises the key barriers under four intersecting themes: stigma and structural violence, gender-based violence, criminalisation, and a lack of female-specific services, and highlights examples of each from Europe. It further analyses the evidence gathered in a qualitative study in Barcelona to determine to what extent barriers are evident in that context.

Barcelona, in Catalonia, Spain, was chosen as the location for this study due to the high formal availability of harm reduction services both in and outside prisons. The city hosts 15 drug consumption rooms and a large network of needle and syringe programmes (NSPs), opioid agonist therapy (OAT) programmes and integrated psychosocial services for people who use drugs. Spain is one of just ten countries worldwide that provide needle and syringe programmes in prisons [32]. This context allows a focus on the unique issues faced by women when accessing those services.

## Methodology

This article comprises a narrative literature review on barriers to access for harm reduction for women who use drugs and the findings of a qualitative study among women who use drugs in Barcelona, Spain.

The literature was sourced from online databases. Using search terms relevant to women and harm reduction and screening by title and abstract, the initial search found 101 papers. Further investigation of these papers and checks for relevant references produced 46 additional papers. Finally, 45 papers were excluded after reading, as they did not bear sufficient relevance to the research question. This left a total of 102 academic papers to be reviewed in this study. This was supplemented with reference to grey literature.

The qualitative portion of this study was completed in collaboration with “Metzineres: Environments of Shelter for Women who Use Drugs Surviving Violence” (referred to as Metzineres), the first integrated harm reduction programme exclusively for women and gender non-conforming people (together participants) in Spain. Metzineres offers direct, holistic and individualised approaches tailored to particular needs, responding to women’s expectations, concerns, curiosities and interests. Throughout it uses an innovative grass roots intervention model sustained by community-based strategies. Its interventions are guided by human rights and gender mainstreaming approaches and invest significant efforts to prove that it is reliable, pragmatic and cost-effective.

Metzineres has a transdisciplinary team, some of whom are survivors of violence or have experience of mental ill health. The team includes a team coordinator and a harm reduction coordinator (both with lived experience of drug use), a doctor, nurse, social educators, social workers, an artist, an administrator and a social anthropologist. They work together with the participants to implement an intersectional feminist, harm reduction and human rights, person-centred, approach founded on the freedom of (re)definition, autonomy, empowerment and improvement of physical, emotional and psychological wellness. Among its goals are to reduce access barriers and increase adherence to health and social care services, through diversified, comprehensive, appropriate, affordable, accessible and high-quality resources and services; uphold women and gender non-conforming people who use drugs as essential interlocutors to influence implementation, development or transformation of public policies and whatever actions that, directly or indirectly, could determine their vital journey and to diminish prejudice, stigma and discrimination against people who use or have used drugs.

In Barcelona, researchers conducted two focus groups, held at the Metzineres harm reduction centre. Staff from Metzineres led engagement with the local community of women who use drugs to invite them to participate. All research participants fulfilled a predetermined set of inclusion criteria, including self-identification as a woman who injects drugs. Researchers also conducted interviews with key informants.

A total of 12 people took part across the two focus groups, and 11 individual interviews were conducted. Among participants were nine people who at the time identified as people who injected drugs and 11 people with experience of incarceration. In addition, three further interviews were carried out with a former prison health worker, a mainstream harm reduction worker and a member of staff at Metzineres.

The focus groups and interviews were held in private spaces, with only participants and facilitators in the room. Both facilitators and all participants signed consent forms and confidentiality agreements. All of the services of Metzineres were available to the participants throughout and after the focus group discussion. The focus group and interviews were semi-structured, following a pre-prepared set of questions but with some flexibility in the amount of time given to each one.

### Stigma and structural violence

Stigma and discrimination are well-documented barriers to health seeking behaviour, engagement in care and adherence to treatment across a range of health conditions globally [11, 33–36]. According to Goffman’s 1963 definition, stigma refers to “negative attitudes and beliefs about certain groups of people. It is the prejudice that comes with labelling and stereotyping an individual as part of a group that is believed to be socially unacceptable.” [37] It leads to status loss and discrimination all occurring in the context of power. Stigma can manifest through health (e.g. disease specific) and non-health (e.g. poverty, gender identity, sexual orientation, migrant status) differences, whether real or perceived. Discrimination is the unfair and unjust action towards an individual or group on the basis of real or perceived status or attributes, a medical condition (e.g. HIV), socioeconomic status, gender, race, sexual identity or age [38].

The barriers women and young girls face regarding access to health facilities include stigma and discrimination from healthcare workers, denial of care, provision of substandard care, physical and verbal abuse, longer waiting periods, passing care off to junior colleagues and disclosure and confidentiality [39]. Current issues that exacerbate this problem include lack of training for healthcare workers on human rights and medical ethics, resource limitations, limited accountability mechanisms and personal moral judgement around culpability. Stigma undermines access to diagnosis, treatment and successful health outcomes.

The stigma of being seen at health services is another major barrier to utilising services. Human rights baseline studies conducted by the Global Fund to Fight AIDS, Tuberculosis and Malaria found breaches in professional ethics by healthcare workers, and a lack of confidentiality and gossiping is common. This was a particular concern for key populations who fear that healthcare workers would disclose their personal information, including their sexual orientation or their HIV status, outside of the clinic setting which would then be known throughout the community. Some providers tend not to refuse treatment but instead make it extremely difficult and more expensive forcing people living with HIV to seek treatment elsewhere [40].

Women’s access to harm reduction services is also hindered by structural violence and stigma. Structural violence is apparent in the greater stigma faced by women who use drugs compared with men, with regard to barriers to harm reduction services for women. Qualitative studies in Georgia, Indonesia, South Africa and Tanzania have consistently found that women face greater stigma based on drug use than men, and that women fear disclosing drug use because of the risk of stigma and social sanctions [41–44]. This has direct consequences on the ability and willingness of women to access harm reduction services. Women are discouraged from accessing services for fear of being identified as a drug user. Not only does stigma dissuade women from accessing services, but it also means women who use drugs can be pushed into hidden and unsafe spaces in order to ensure that their drug use is not made public [41–44].

Women who use drugs experience generalised social stigma and direct stigma and discrimination from health professionals, including those involved in providing harm reduction services. As in the wider public, this stigma is more acute for women than men because of social expectations about womanhood and the role of women. Women have reported pervasive stigma across the health system in studies conducted around the world [4, 41, 42, 45–47]. For example, it is common for women in the UK to face open gender- and drug use-related stigma and discrimination when accessing opioid agonist therapy, particularly in pharmacies [4].

A systematic review of stigma towards people who use drugs from health professionals found that negative attitudes are pervasive and that they lead people who use drugs to avoid health and harm reduction services [26]. Experienced stigma leads to the anticipation of stigma, which discourages people who use drugs—and particularly women who use drugs and face greater stigma—from accessing services.

Female sex workers who use drugs are subject to even greater stigma, and are more vulnerable to harmful consequences. These women are more likely to work in less safe conditions (for example street-level sex work and exclusion from brothels) than their colleagues who do not use drugs and as a result are more likely to experience violence and higher-risk sex [16, 18, 48–50]. These conditions, combined in many cases with punitive legal and policy environments for people in the sex industry, reduce their ability to access harm reduction services.

Lowering thresholds for service access and making service provision more discreet and flexible can help diminish the discouraging effect of social stigma. This may include providing greater quantities of equipment in needle and syringe programmes to minimise the number of visits necessary, having flexible opening hours, assuring confidentiality, providing services in pharmacies and permitting secondary syringe distribution [16].

In Barcelona, women who use drugs reported stigma to be a pervasive experience in their lives, and an experience that leads to deprioritising self-care and harm reduction. One women who uses heroin and crack daily said:

Today I had money, and I wanted to spend it on something nice for me. I went to a hairdresser and she look at me with rejection. She didn’t want to do my hair. Why? I am clean, I am cleaner that most of her clients. Finally, I expended my money on crack.

Many women and gender non-conforming people attending the Metzineres centre no longer access the mainstream harm reduction, health and social services that are available in Barcelona. They report that this is a result of multiple interrelated factors that drive social exclusion and stigma, among which are drug use, sex work, migration status, gender identity and living with HIV and/or hepatitis C. The stigma and discrimination they experience are mutually reinforcing with wider experiences of structural violence: extreme poverty, homelessness, family breakdown and the loss of custody of their children.

One of the central goals of Metzineres has been to challenge the stigma and structural violence experienced by women and gender non-conforming people who use drugs, and for them to be considered (and consider themselves) a part of the community rather than challenge the community must face. Many participants reported that Metzineres is the first place that they feel safe, with the power to define their own engagement, according to their personal circumstances, well-being and levels of trust. They are not service recipients, but experts who drive the services provided at their own pace. Staff at the centre aim to mitigate any perception of failure, to avoid re-victimisation and secondary traumatisation, while also recovering power, community and belonging. One participant, a transwoman with experience of migration, reported that:

It is the first time that before hiring someone the staff ask for our opinion. We have an assembly every Wednesday where we decide what activities we want or if there are any problems. We discuss a lot, and we are not always agreed, in fact, most of the time we are not, but it doesn’t matter because it is ours. And we never had anything like that before. We are a dysfunctional family but a family who sticks together to face the problems together.

Creating active participation in the community is one of the main goals of Metzineres. They facilitate the participation of Metzineres participants in wider society in contexts free of stigma and discrimination. This includes organising and participating in social events in communal spaces in the El Raval district. Metzineres and the participants who frequent the centre have substantial links with other social, communitarian and solidarity movements and organisations in the area and have faced very little opposition from local actors. As reported by one woman experiencing homelessness:

You know, one of the things that happen when you are on the streets, shooting every day, is that you just talk with other drug users or service providers. But [Metzineres] is not like that. We have our paella on Fridays and a lot of different people come to have lunch with us. Now I can go to the community garden and they are my friends. It is nice to meet people outside of this world sometimes.

One woman who injects methamphetamine emphasised the effect of how such an environment helps to confront self-stigma and promote self-belief and empowerment:

When a friend of mine told me about this place I couldn’t believe her. First weeks coming here I didn’t know who was staff and who wasn’t. I thought that I deserved everything that happened to me. Now I know that we have rights and if we are together, we can accomplish a lot of things.

Another participant, a transwoman with experience of sex work and migration, also highlighted the way in which engagement with Metzineres had addressed self-stigma:

I don’t feel ashamed anymore, now I have an example of how I want to be treated. I have the right.

### Gender-based violence

The World Health Organization has found that one in three women will experience violence in her life, most commonly at the hands of an intimate partner [51]. Violence against women and girls is one of the most prevalent human rights violations in the world. It undermines the health, dignity, security and autonomy of its victims [51]. Gender-based violence knows no social or economic boundaries and affects women and girls of all backgrounds [51].

Women and girls who are survivors of violence suffer a range of health consequences, including mental health issues such as depression and anxiety, higher use of alcohol, less control over sexual decision-making and poor sexual and reproductive health outcomes. In some regions, women and girls who have suffered intimate partner violence are 1.5 times more likely to acquire HIV than women who have not suffered such violence [52]. Violence against women is estimated to cost countries up to 3.7% of their gross domestic product—more than double what most governments spend on education in some countries [53].

A study in Spain found that a high proportion of women who use drugs suffer psychological or physical violence by partners. Results from the study show that 88% of women who use drugs reported having ever suffered emotional or psychological damage, 71% indicated having experienced at least one incident of serious physical injury by a male partner and 49% having ever suffered sexual abuse. Similar to other studies, results show the syndemic of substance abuse, partners violence, HIV, mental illness and social instability [54]. In comparison with men, women who use drugs have considerably more frequent and intense experiences with interpersonal violence, sexual abuse and trauma.

For women who use drugs, the violence they face has become a major public health problem. In many countries, police are often enforcers of the war on drugs and the first responders to reports of domestic violence. As a result, women who use drugs are often treated as potential offenders, rather than as people whose health and rights they are charged with protecting.

Most services related to health care, gender-based violence and other social issues are not integrated. Women who experience gender-based violence are sent from one service to another to address different issues, and this lack of integrated service delivery is another deterrent for women accessing any services [42, 43, 55]. Drug use and intimate partner violence are defined by power relations based on gender, race and class [56]. As such, it is vital that services providing health and harm reduction services to women who use drugs are able to respond to the needs of those who have experienced intimate partner violence [57].

However, existing research shows that service staff are rarely equipped to meet those needs. A study among staff in Canada found that when accessing health and harm reduction services, people with experience of intimate partner violence encounter stigmatising or victim-blaming attitudes [55]. Under-resourced staff, both in terms of training and high caseloads, reduces the likelihood that intimate partner violence will be addressed [58]. The lack of services for people with experience of intimate partner violence is an unmet need in harm reduction services.

Links between drug use and intimate partner violence have been established in systematic reviews, which find that they can in some cases be co-occurring mutually enhancing phenomena, influenced by power relations based on class, race and gender [56]. The exact nature of the link is unclear [59]; however, there is a wealth of evidence that intimate partner violence has a negative effect on access to harm reduction services for women who use drugs. While physical assault by a husband, boyfriend or former partner is generally associated with increased access to health services (because of the occurrence of physical injury and mental trauma), the same is not true for women who use drugs [60].

Intimate partner violence affects women’s autonomy in accessing harm reduction services in cases where an abusive partner obstructs access. Multiple studies among women who use drugs and service staff have found that abusive partners deliberately prevent women from accessing harm reduction services through violence, threats and other abusive behaviours [58, 61, 62]. Women living with HIV or hepatitis C may not access treatment for fear of disclosing their serostatus in the knowledge that doing so might incite intimate partner violence [63]. As a result of these influences, intimate partner violence is a major cause of lack of engagement in, and attrition from, harm reduction programmes.

Many of the women at the Metzineres centre in Barcelona reported lived experiences of violence. This can be during adulthood and/or childhood, from partners, family members, other acquaintances and/or strangers. Staff and participants at Metzineres report a severe lack of public services able to meet the complex needs of women and non-gender conforming people who use drugs and have experience of violence. As identified elsewhere in Europe, these experiences are commonly addressed separately by mainstream services without acknowledgement of the complex interdependence between the two. One member of staff at Metzineres reported that:

Services related to women surviving violence are not an answer. They do not accept women who use drugs, or they have conditions that are incompatible with the situation of most of them.

Equally, according to the same staff member, mainstream harm reduction services are incapable of addressing the needs of women with experience of violence:

They do not take into account their specificities, their experiences of trauma and needs of safety and protection particularities or their strategies to cope with them.

As a result, women report resorting to unofficial and sometimes illegal means to protect themselves from abuse. *Narcopisos,* apartments (often squats or under illegal occupation) where people can buy and use drugs in private, emerged as an alternative to the official drug consumption spaces. One women reported that she had escaped an abusive relationship by setting up a *narcopiso*:

I was the first who opened a narcopiso.[…] I had a fight with my ex-husband. He told me that if I continue dealing he would kill me. I was in a squat with a friend and I told her to bring people because I was hiding and couldn’t go out. It was successful for a long time because cops did not know what it was or what was going on. I spent 6 years in jail and when I was released everybody was doing the same.

While for some women such spaces may offer shelter from law enforcement and abusers, focus group participants commonly reported sexual and physical assault that occurs in the *narcopisos*. One participant described how reliance on these spaces can make women more vulnerable to violence:

The narcopisos have been through a huge change, there are strong mafias behind, they don’t live in the neighbourhood. They just came here because there were empty flats where they can open their business for free, day and night. They control all the market. I have to buy from them.[…] They don’t give a shit for the clients or the neighbours and the level of violence has risen. What women suffer in that places is terrible.

There is a clear need for services that understand the complex needs of women who use drugs and have experience of violence, without forcing them into hidden spaces where they are at greater risk of social, physical and drug-related harm. Metzineres seeks to provide such a space. As one women experiencing homelessness and an abusive partner reported:

Coming here I could break my relationship with him. He hurt me, but I was afraid of sleeping in the streets by myself. Now I know that I am not alone anymore, we have our strategies to protect each other.

### Criminalisation

Criminalisation creates a significant barrier to harm reduction service access among women who use drugs in Europe, by dissuading service engagement for fear of exposure and by providing police with an incentive and opportunity to obstruct harm reduction behaviours. The harsh policing associated with criminalisation can diminish the agency of people who use drugs to manage their drug use and reduce harm [64]. The impact of criminalisation on barriers to harm reduction services for women who use drugs can be broken into three interconnected phenomena:

- formal, legal barriers that obstruct access for women who use drugs;
- interactions with law enforcement agencies that reduce the ability or willingness of women to access services; and
- the incarceration of women who use drugs.

In many cases, women face formal, legal barriers to harm reduction services that are not faced by men, at least not to the same extent. Key among these barriers are those placed in the way of pregnant or parenting women. Examples of this include clinical restrictions placed on women’s access to harm reduction services: for example, in Denmark pregnant women are the only group explicitly excluded from accessing drug consumption rooms, because of potential foetal harm, despite the greater potential harm of unsafe injection [65, 66]. In other cases, women can face criminal charges or a loss of parental rights for drug use while pregnant or parenting. These circumstances are not proven to deter drug use, but do imperil the relationship between women and health providers [65]. The fear that children will be removed from their mother’s care if drug use is disclosed, or in some jurisdictions including in Russia and Ukraine, the risk of prosecution for child abuse or murder creates an even stronger deterrent to these women accessing harm reduction services [14, 16, 18, 21, 24, 67].

This deterrent effect is enhanced where service providers are obliged to report drug use to law enforcement agencies or social services, or where a registry is kept of people accessing harm reduction and drug services (i.e. obligatory registration for service use) [65]. Such regimes are relatively common in Eastern Europe, and inclusion in such a registry can have far-reaching effects, including ineligibility for antiretroviral therapy and housing programmes, as well as loss of parental rights [16, 48, 68]. The cumulative effect of this environment, driven by the criminalisation of drug use and people who use drugs, is to intensify the barriers that women face when accessing harm reduction and health services.

The criminalisation of drug use by its nature also drives greater interactions between women who use drugs and law enforcement officers. Drug use can be exploited by law enforcement officers as justification for abuse, with aggressive policing techniques that include arresting women for carrying injection or smoking equipment, planting drugs, harassment, soliciting bribes, sexual abuse and violence [68]. By giving law enforcement licence to arrest and threaten women who use drugs, criminalisation pushes women into more hidden spaces in order to avoid such interactions [69].

Accordingly, research finds that women who use drugs, and particularly female sex workers who use drugs, face harassment (including frequent arrest, confiscation of possessions, blackmail) and violence (including sexual violence) at the hands of law enforcement officers [48, 68]. This has a direct impact on their ability and willingness to access harm reduction services or to be reached by outreach teams [18, 70].

The number of women incarcerated worldwide has increased by 53% since 2000, [71] and substance use is clearly present in women’s prisons [17]. A higher proportion of women than men are incarcerated for drug-related offences [72]; for example in Europe and Central Asia, an estimated one in four female prisoners has been convicted of a drug offence [17]. Sex workers who use drugs are particularly vulnerable, with the dual criminalisation of sex work and drug possession putting them at particularly high risk of incarceration [17].

Access to harm reduction in prisons is severely limited worldwide, and there is a grave dearth of data on prisoner health [73, 74]. HIV, viral hepatitis and tuberculosis treatment and prevention, including NSPs and OAT, are near universally less accessible in prison that outside [13, 74]. As a result, incarceration represents a significant risk factor for blood-borne virus transmission associated with injecting drugs [68, 74].

Despite the growing population of incarcerated women, antiretroviral therapy for HIV treatment, NSPs and OAT are all more widely available in male prisons than in female prisons [68, 74]. Men are consistently prioritised for prison health services, due to the larger number of men incarcerated and therefore the greater urgency and cost-effectiveness of providing services to male prisoners [75–77].

Women consistently report unsafe injection behaviour in prison in the absence of accessible sterile injecting equipment [13, 78–80]. This includes syringe sharing, the use of improvised injection equipment, and the use of bleach as disinfectant [13, 80–82]. Women in prisons where harm reduction services are available can face the same barriers to access as women outside. Women report that stigma persists around drug use and HIV, which can discourage engagement [13]. Finally, incarceration has a disproportionate effect on women’s access to antiretroviral therapy compared with men, due to the shorter sentences typically served by women and the challenges of continuing care between prison and the community [13].

In Barcelona, women reported all three of these barriers to harm reduction services. Multiple participants reported separation from their children and rejection from programmes for women who have experienced violence due to drug use and/or incarceration. Once their children had been removed from their care, several women reported difficulties in having them returned after release from prison. Social exclusion driven by the physical isolation of being in prison and discrimination against women who use drugs or women who have been incarcerated prevent them from meeting the administrative requirements necessary to take on care of their children. This was expressed by two female participants:

Do you think that I wouldn’t prefer a regular job? But I haven’t had a contract for over 20 years and I am a mum with 3 kids. Two of them are in a governmental juvenile facility because they consider that I don’t have enough money to provide for them. They think that my children are better in a centre that with their mother. I love them so much, and they love me too. They want to stay with me all the time, when they finish school they come for a visit, and we are together for the weekend.

When I was released from jail after 6 years for trafficking everything was different. My community was gone and I was alone. I tried to find a job, but who would give me a job, with my history of incarceration and no experience… I needed money to be able to recover my kids.

Interactions with law enforcement officers, notably being targeted for searches, were also commonly reported. In one case, this referred specifically to interrupting a community organisation of women who use drugs:

We were leaving from the XADUD [Catalan Network of Women who Use Drugs], where we were organizing for the feminist demonstration and cops stopped and searched us. We try to explain, and I think that they knew that we did not have anything stolen, for sure they knew that we were not selling. Anyway, I think that they were just bored.

While services such as NSPs and OAT are generally available in Spanish prisons, including those in Catalonia, women reported that incarceration still stood as a significant barrier to accessing such services. One former prison medical worker expressed concern that restrictions on access to harm reduction in prison were greater for women than for men:

Women are made invisible, with the excuse that there are fewer of them – only 4% of the prison population. So, their needs are ignored.

With regard to the NSPs in prisons, participants reported that the existence of such a programme does not mean that people will use it. Barriers include concerns about poor quality, insufficient or inappropriate equipment given out by the NSPs:

The kit they give you as alcohol wipes and a syringe, but where are you supposed to cook the substance, in a spoon? They don’t give you a cooker, they don’t give you basic paraphernalia. The secondary complications of cooking the substance in something unsterile are serious.

As has been documented in prison NSPs elsewhere (such as in Canada [83]), a lack of anonymity can result in punitive responses from prison security staff:

Once you go and request a syringe they don’t leave you alone. They follow you, because they know you’re going to inject. They know you’re going to inject, so they watch you, they search for you.

They hassle you. They look for the substance you’re going to consume.[…] The NSP should be free from consequences like that, it should be anonymous and you shouldn’t have to be hassled once you’ve used it. Because it’s true that a lot of women stop going to it and just use whatever type of utensil or paraphernalia, which isn’t appropriate.

In a finding not noted elsewhere in the literature, women reported that while OAT is widely available, it is commonly misused by prison authorities. Several women mentioned overmedication of methadone, as well as benzodiazepines:

When I went in, they kept giving me methadone and pills. They give them to you quite happily. There’s brutal overmedication.

### Female-centred services

The effects of entrenched gender inequities and norms are apparent in the services available in harm reduction facilities and organisations [18, 84, 85]. Harm reduction services in Europe remain overwhelmingly gender blind or—more commonly—male focused. El-Bassel and Strathdee relate this to a “masculinist” tendency in harm reduction service provision, in which services are designed primarily by and for men [68]. As a result, in many cases harm reduction services are masculine spaces not tailored to the needs of women.

Women’s health decisions, including those relating to drug use, do not take place within a void, but are syndemic and influenced by, and limited by, environmental, social and economic factors [17, 86]. Gendered power inequities in society can reduce women’s autonomy in accessing harm reduction and HIV prevention services and practices, in turn increasing their risk of HIV acquisition [87, 88].

In combination, these factors leave women underserved by harm reduction services and the specific issues they face poorly understood.

In Europe, concentrated efforts by local leaders have resulted in a number of strong, but niche examples of services designed to be sensitive to gender. More broadly, women experience common social and structural barriers across the region. The overall effect of this is that women are consistently reported to be at higher risk of HIV and hepatitis C infection than men who use drugs, and have greater vulnerability to the harms of drug use. For example, Switzerland has been successful in substantially reducing the number of drug-related deaths (largely attributed to opioids) since 1995; the overall number of drug-related deaths fell by 64% from 1995 to 2016 (from 376 to 136) [89]. However, the decline in drug-related deaths during this period among women (51%) was less pronounced than the decline among men (68%) [90, 91].

Concentrated efforts have resulted in services for women who inject drugs in a number of countries around Europe. Examples of this include an NSP for women who inject drugs in Malta [92] and a syringe dispensing machine in a women’s prison in Germany (a syringe-dispensing machine in one out of 181 prisons—a woman’s prison with 200 inmates) [32, 93].

Pregnancy and reproductive health is a key motivator for women who use drugs to engage in harm reduction services that are relevant to their needs. Where these services are absent from harm reduction, it can form another barrier to access for women. Where services have been integrated, evidence shows that uptake of both reproductive health and harm reduction services is increased. By either sharing a site or having robust referral pathways, reproductive health and harm reduction services can be mutually reinforcing in reducing barriers to access [8].

Peer networks can play an important role in supporting women’s to access services. In Europe, there are two formally established networks for women who use drugs in Italy (the Chemical Sisters) and in Spain (XADUD—*Xarxa de Dones que Usen Drogues*, Network of Woman Who Use Drugs) which is connected to CATNPUD, the Catalan network of people who use drugs [32].

Stigma, self-stigma and criminalisation all contribute to lower testing and access to treatment among people who inject drugs than the general population; women (as well as migrants and people in rural areas) are reported to face compounded barriers [94].

Women are reported to face more restrictions than men, including hostile and judgemental attitudes from health professionals [94, 95]. With responsibility for parenting disproportionately falling to women, harm reduction services that do not meet the needs of mothers represent a significant barrier to access for women who use drugs. Firstly, women consistently report that a lack of childcare facilities means they are unable or unwilling to access harm reduction services [16, 19, 43, 45, 67, 96]. In some cases, this is reported as the most significant barrier to engagement [16]. For example, a lack of childcare at OAT services represents a barrier to engagement [94, 95]. Secondly, women’s parenting obligations also mean that they may not be able to access services during fixed operating hours or at fixed intervals, underlining the importance of flexible services in providing for women’s participation [97, 98]. Thirdly, mothers who use drugs report that they are reluctant to access health and harm reduction services because of the risk of losing custody of children based on drug use [81, 99–101].

A consistent concern about women’s access to harm reduction services in Europe and globally is that women are often required to navigate multiple separate health and referral systems to address an interrelated health and social concerns. For example, though sexual and reproductive health, mental health, intimate partner violence and drug use can be co-occurring phenomena, it is rare that women can get holistic support across these issues [42]. Women in Georgia report that the lack of services for people with experience of intimate partner violence is an unmet need in harm reduction services [43]. Depression, anxiety and post-traumatic stress disorder, in some cases related to physical or sexual abuse, have been found to be more prevalent in women who use drugs than in men in studies in Spain and globally [60, 102–105]. Where services addressing these issues are not available, women are deterred from accessing or are less likely to adhere to harm reduction programmes such as opioid substitution therapy [106]. By ensuring that services are integrated, either sharing premises or with strong referral pathways, it can be ensured that women have access to the harm reduction services they require [19].

Services that protect women’s privacy and safety are necessary to ensure that harm reduction is accessible to women who use drugs. Women-only spaces and services help to guarantee the personal safety of women, reduce the impact of imbalanced gender power dynamics and improve health outcomes [8]. Additionally, including women in the design and operation of harm reduction services increases awareness of women’s needs.

By providing a space only accessible to women and non-gender conforming people, Metzineres creates an environment for healing, training and recovery that addresses the challenges its participants face holistically. Through the physical space in El Raval and connections with other local social movements, networks and organisations, the organisation aims to meet basic needs, give social health care and provide a range of services and activities for healing, bonding, experience and wisdom sharing, self-defence, solidarity and mutual support. Focus group participants expressed the benefits of having access to a safe space, without any requirement to participate in any specific activity:

I come here every day, inject my dope safely, clean my clothes, sleep for a few hours knowing that nothing bad is going to happen, have something to eat, see a female doctor and participate in a workshop where I can make my own shampoo. And if I don’t want to do any of these, I can be just having a coffee with my friends [amigas], being some of them staff members.

It is the only place that I am allow to stay with my dog, he is my partner, I don’t want to leave him outside. It’s funny, they allow dogs but not men.

Recognition of and solidarity with the common challenges and experiences of women who use drugs is a crucial part of the mission of Metzineres, shared by the women who attend the centre. In February 2019, Metzineres organised a meeting in Barcelona with the Eurasian Harm Reduction Association and the Association for Women’s Rights in Development and also invited other groups such as the International Network of Women who Use Drugs, the Women and Harm Reduction International Network and the European Network of People who Use Drugs. The meeting shared experiences and best practices and resulted in the Barcelona Declaration, recognising the gendered aspects of drug policy, and the disproportionate harms woman have to suffer as a consequence of structural inequalities. One focus group participant shared her experience of engaging in this forum:

It was amazing meeting women from around the world that have similar lives and problems than us. We could do more things together. It is hard to know that they cannot have access to methadone, as we do. Now we have the responsibility to fight not just for us but for our friends’ rights also.

## Conclusions

The evidence in the literature on barriers to accessing harm reduction for women who use drugs can be broadly categorised in four intersecting themes: stigma and structural violence, gender-based violence, criminalisation and a lack of female-specific services.

Stigma, whether experienced or anticipated, reduces women’s willingness to access harm reduction services. While all people who use drugs face stigma based on drug use, cultural norms around womanhood mean that women who use drugs are doubly stigmatised. Generalised stigma in society prevents women from accessing harm reduction services for fear of their drug use becoming publicly known. This can push women into more hidden spaces, making it less likely that they will be approached by outreach workers. Stigma experienced or expected from service staff dissuades women further from accessing services. Self-stigma—a result of gender expectations and experienced stigma—compounds this effect by reducing health-seeking behaviours and therefore access to harm reduction.

Gender-based violence can stifle women and gender non-conforming people’s autonomy and encourages those at risk of violence to deprioritise harm reduction practices. In cases where women are at an immediate risk of violence, this can be a rational response to different threats to health. Few social services are willing or capable to address gender-based violence and drug use as co-occurring and interrelated phenomena, meaning that women and gender non-conforming people are often left out of one or both.

The criminalisation of women and gender non-conforming people who use drugs drives them away from formal services and towards less safe patterns of use. This is a result of several factors. Firstly, formal barriers prevent certain women from accessing certain services, such as restrictions on services for pregnant or parenting women. Obligatory registration in harm reduction services can also deter women, again particularly those who are pregnant or parenting, from accessing services. Secondly, experiences of arrest, harassment and violence by law enforcement officers are particularly acute for women and gender non-conforming people who use drugs, and can push them into more hidden spaces and away from mainstream harm reduction services. Finally, the inequivalence of harm reduction service access between prisons and the outside community means that the incarceration of women who use drugs places them in an environment with no access to harm reduction services. Where services are available in prisons, willingness to access such services is commonly lower than outside due to controls on access, even greater breaches of confidentiality and stigma.

Few harm reduction services are designed specifically with women and gender non-conforming people in mind. As a result, they commonly are poorly integrated with services to address the needs of these populations, notably sexual and reproductive health services, services for people who have experienced gender-based violence, and childcare. This lack of services reinforces the perception that harm reduction facilities are masculine spaces, thus discouraging access by those who do not identify as male. Furthermore, the relationships between barriers and access, such as gender-based violence, stigma, criminalisation and unspecialised services, are poorly recognised and under-addressed in health and harm reduction responses.

Women who use drugs in Barcelona experience all of these barriers. However, their testimonies demonstrate that the existence of Metzineres as a service specific tailored and responsive to their needs goes some way to mitigate these barriers. By creating an environment in which the principles of harm reduction, social inclusion, human rights and gender responsiveness are emphasised, Metzineres challenges the marginalisation faced by many of its participants. The approach enables Metzineres to provide holistic and personalised care for those who access its services. As reported by the participants in this research, the safe space at Metzineres enables women to share experiences and create a sense of solidarity, intensifying the service’s ability to combat stigma and self-stigma.

However, there are limits to what a service such as Metzineres can achieve. Many of the barriers to harm reduction faced by women and gender non-conforming people who use drugs are structural and can only be addressed through widespread policy and societal change. This includes criminalisation and incarceration, stigma in society, and the legal status and availability of harm reduction interventions. In this respect, the way in which Metzineres and its participants engage in political activism and with the wider social movements in Barcelona and internationally is crucial to understanding its role in increasing access to harm reduction. This is exemplified by the creation of the Barcelona Declaration and the active participation of Metzineres participants in that process.

Enabling access to harm reduction services is essential in order for states to meet their human rights obligations. The estimated 3.2 million women worldwide who inject drugs must have equitable access to harm reduction. While services such as Metzineres can be life changing and life affirming for the women they serve, it is incumbent on states to act to address the structural barriers to health faced by women who use drugs.

## Data Availability

Data sharing is not applicable to this article as no datasets were generated or analysed during the current study.

## Abbreviations

EMCDDA: European Monitoring Centre for Drugs and Drug Addiction
HIV: Human immunodeficiency virus
NSP: Needle and syringe programme
OAT: Opioid agonist therapy

## Footnotes

1 EMCDDA refers to this group as people with “serious drug problems”. The authors have chosen to use the term “people with drug dependence” following the latest language guidance from the International Network of People who Use Drugs and the Asian Network of People who Use Drugs [9].
